# Specific branches of hypoglossal nerve to genioglossus muscle as a potential target of selective neurostimulation in obstructive sleep apnea: anatomical and morphometric study

**DOI:** 10.1007/s00276-016-1778-7

**Published:** 2016-11-17

**Authors:** Philippe Delaey, Jérôme Duisit, Catherine Behets, Thierry Duprez, Pierre Gianello, Benoît Lengelé

**Affiliations:** 1 Pôle de Morphologie (MORF), Institut de Recherche Expérimentale et Clinique (IREC), Université Catholique de Louvain, Avenue E. Mounier 52, Bte B1.52.04, 1200 Brussels, Belgium; 2 Pôle de Chirurgie Expérimentale et Transplantation (CHEX), Institut de Recherche Expérimentale et Clinique (IREC), Université Catholique de Louvain, Avenue Hippocrate 55 bte B1.55.04, 1200 Brussels, Belgium; 3 Department of Plastic and Reconstructive Surgery, Université Catholique de Louvain, Cliniques Universitaires St-Luc, Avenue Hippocrate 10, 1200 Brussels, Belgium; 4 Department of Radiology and Medical Imaging, Université Catholique de Louvain, Cliniques Universitaires Saint-Luc, Avenue Hippocrate 10, 1200 Brussels, Belgium

**Keywords:** Obstructive sleep apnea therapy, Supra-selective neurostimulation, Hypoglossal nerve, Genioglossus muscle

## Abstract

**Purpose:**

To determine the ideal implantation site for selective tongue neurostimulation in obstructive sleep apnea, anatomy of the distal branching of the hypoglossal nerve (HGN) was revisited.

**Methods:**

The HGN distal course and intramuscular distribution to the tongue muscles were studied in 17 embalmed and 5 fresh heads (age 60–98, BMI 20–35). Medial branches supplying selectively the genioglossus (GG) muscle were identified. Then, the distinct bundles entering the oblique (GGo) and horizontal (GGh) parts of the GG were located. Morphometric data were compared to similar measurements made on MRI sections from 12 patients (age 43–71, BMI 18–47).

**Results:**

The key facts relevant to optimize stimulation and electrode design are the following: the mean width of both GG muscles in embalmed and fresh cadavers was 20.7 ± 2.9 and 21.4 ± 5 mm, respectively; it is significantly (*p* < 0.05) superior to the MRI value of 18.26 ± 2.0 mm. Selective nervous branches for GGh and GGo were located at 52 ± 8% of hyoid bone-mandibular symphysis distance and at 5.8 ± 1.1 mm from the inferior border of the GG muscle. The surface of stimulation is a 4.4 ± 1.1 × 6.9 ± 3.8 mm ellipse.

**Conclusions:**

According to our observations, the optimal selective or supra-selective stimulation of the tongue protractor muscles can be performed on the lateral surface of the GG at roughly equal distance between the mandibular symphysis and the hyoid bone, at a depth of about 0.6 cm above the GG lower border.

## Introduction

Obstructive sleep apnea (OSA) is a chronic condition due to periodic collapse of the pharynx during sleeping. This airway obstruction prevents normal breathing and results in oxygen desaturation, micro-arousals with sleep fragmentation and abnormal sympathetic activation [2, 6]. Severe OSA, characterized by more than 30 apneas-hypopneas per hour, leads to non-restorative sleep with excessive daytime sleepiness, increased cardiovascular morbidity and mortality, and both higher stroke risk and all-cause mortality [9, 22, 23]. Such complications can be prevented by early detection and effective interventions. The standard treatment of OSA consists in application of a continuous positive airway pressure (CPAP). Despite its proven efficacy [13], this symptomatic treatment shows major inconveniences due to the cumbersome apparatus and noise generated. At the end, up to 40% of subjects suffering from severe OSA do not comply with CPAP, and therefore remain untreated [5]. In the recent years, devices providing neurostimulation of the hypoglossal nerve (HGN) were developed as an alternative for noncompliant CPAP patients [4, 11, 21]. Relying on tongue protraction to open pharyngeal airway, they appear to be efficient in preventing apnea [1, 18]. Moreover, a potential positive residual effect was observed in patients using those neurostimulators for a long time, suggesting a disease modifying effect [14].

HGN neurostimulation efficiency depends on targeted segment. Stimulation of the proximal truncular segment which supplies both intrinsic and extrinsic tongue musculature [4, 11] is nonselective, and thus less effective. A more distal stimulation of the protrusive muscles [21] is more efficient. As detailed by Mu et al. [10], HGN splits into medial (m-HGN) and lateral (l-HGN) contingents. The m-HGN branches are preferentially distributed to the genioglossus (GG): they favor tongue protraction, and consequently oropharyngeal airway dilatation [12, 16]. Consequently, they represent a highly valuable selective target for all therapeutic neurostimulators. The GG muscle can be divided in two morphological and functional compartments, according to fibers distribution, action and nerve supply [10, 12]. The first, oblique compartment (GGo) is constituted of vertical fibers which contraction depresses the tongue without affecting pharyngeal dimensions. The second, horizontal compartment (GGh) contains longitudinal fibers protruding the posterior part of the tongue and enlarging the pharyngeal cavity. As a matter of fact, the supra-selective stimulation of GGh fibers should be the ultimate goal in OSA treatment.

Our anatomical study focused on the HGN distal course to locate the terminal ramifications supplying the GGh and GGo compartments, and to optimize the implantation of neuromuscular stimulation devices. Morphometric data were obtained from cadaveric dissection and in vivo MRI, aiming to determine the feasibility of a supra-selective m-HGN/GGh stimulation.

## Materials and methods

### Anatomical study

#### Specimens

The hypoglossal nerve was dissected bilaterally on 17 embalmed adult cadavers (7 men and 10 women). This dissection was also performed on 5 fresh heads (5 men) to identify possible postmortem or fixation variations which could influence morphometric data. These subjects were 60- to 98-year-old and their mean BMI was estimated to be at 27.3 ± 3.4 (range 20–35). To precisely determine the neurovascular relationship, common carotid arteries of one fresh head were cannulated and injected with blue-colored latex, later polymerized with acetic acid. The head was kept overnight at 4 °C and then frozen at −20 °C, to be finally thawed at room temperature prior to proceeding.

#### Nerve dissection

The hypoglossal nerve (HGN) was identified in the submandibular region, at the level of the greater cornu of the hyoid bone, laterally to the hyoglossus (HG) muscle. The distance between the mandible and the hyoid bone was measured before any dissection. The median cleft between the two geniohyoid (GH) muscles was split to reach the GG muscles; they can easily be identified by their interposed fatty raphe. Mandibular insertions of both GH muscles were then transected to expose the GG muscles. Under 4.5× magnification loupe, HGN dissection was performed to identify its first selective split into lateral (l-HGN) and medial (m-HGN) contingents. The m-HGN was then followed until its supra-selective branches, respectively, to the horizontal (GGh) and oblique (GGo) compartments of the GG muscle. These supra-selective branches were dissected down until their muscular penetration. The number of HGN branches supplying HG muscles was assessed bilaterally in 9 cadavers (*n* = 18). The number of HGN branches supplying GH and GG muscles was evaluated on both sides in 15 cadavers (*n* = 30).

#### Morphometry

**Fig. 1:**
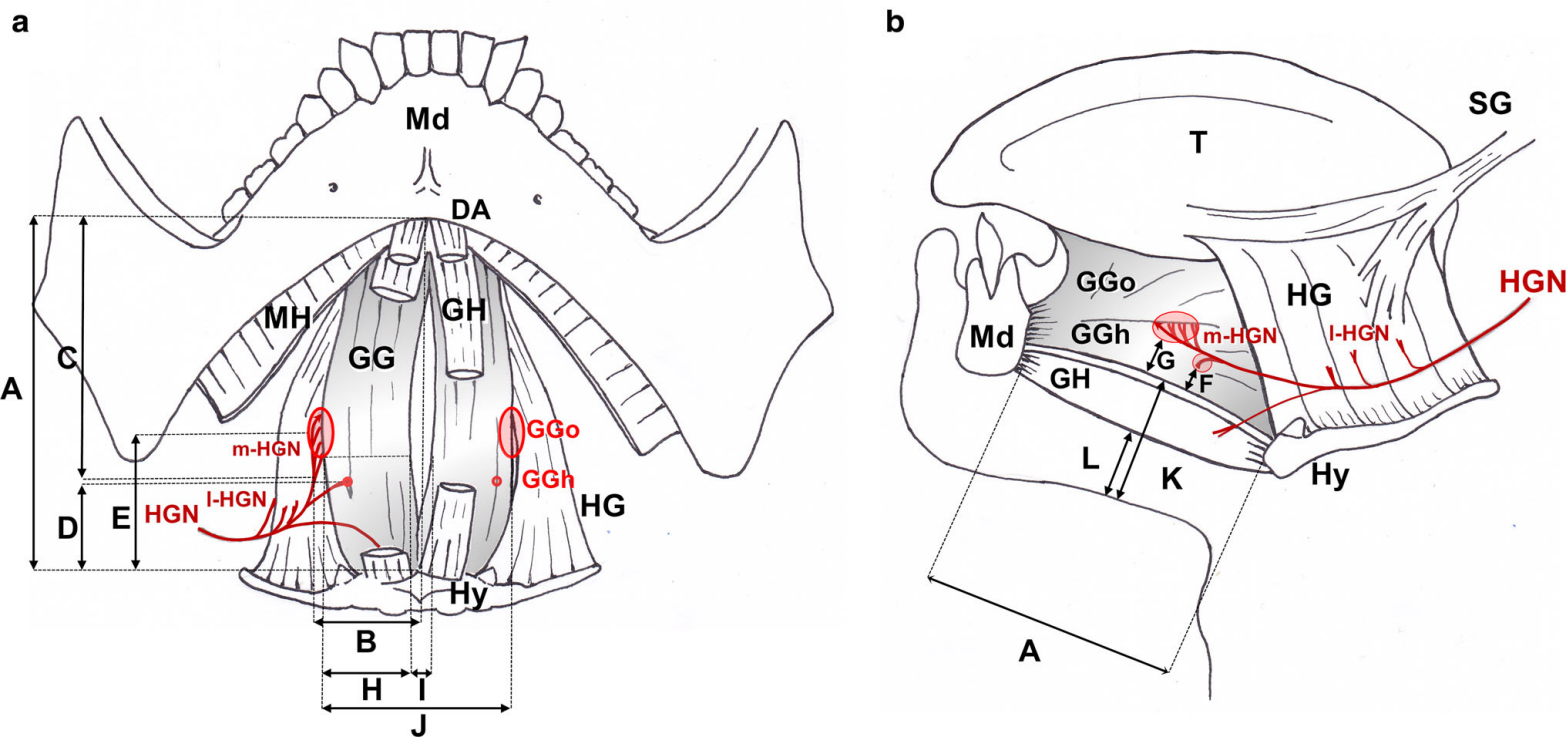
Schematic landmarks for anatomical measurements under study. **a** Antero-inferior view. **b** Lateral view. Anatomical structures are labeled as follows: mandible (*Md*), hyoid bone (*Hy*), hypoglossal nerve (*HGN*), lateral contingent of the distal hypoglossal nerve (*l-HGN*), medial contingent of the distal hypoglossal nerve (*m-HGN*), genioglossus muscle (*GG*), horizontal fibers of GG (*GGh*), oblique fibers of GG (*GGo*), digastric anterior belly (*DA*), mylohyoid (*MH*), geniohyoid (*GH*), hyoglossus (*HG*), styloglossus (*SG*), tongue intrinsic muscles (*T*)

Based on reliable anatomical landmarks, the following distances were measured to describe the selective nervous supply and the dimensions of the GG muscle (Fig. 1a, b): distance *A*, located on the midline, from the mandibular symphysis to the anterior median crest of the hyoid body; distance *B*, from the point where the HGN crosses the HG anterior edge, perpendicularly to the midline; distance *C*: from the HGN selective branches for GG muscle to the mandibular symphysis; distance *D*: from the supra-selective GGh branches to the hyoid; distance *E*: from the supra-selective GGo branches to the hyoid (lesser cornu); distance *F*: depth of selective GGh branches to the inferior surface of GG; distance *G*: depth of GGo terminal branches to the inferior surface of GG; distance *H*: transverse width of a single GG, where the nerve branches entered it; distance *I*: width of the median fatty raphe; distance *J*: width of both GG muscles at the nerve junctions area; distance *K*: skin to GG inferior surface, measured at equal distance between the mandible and hyoid bone; distance *L*: skin to GH inferior surface measured at the same point.

#### Delineation of HGN selective stimulation area

To define a probabilistic area for selective GG stimulation, we calculated a mean central point and its related area for branches location measured in the cadaver study: to obtain a relative distance in the anteroposterior axis, distances *D* and *E* were divided by distance *A*, then expressed as a percentage (%) of the geniohyoid distance from hyoid bone. Based on these results, an associated area was calculated, expressed in vertical axis × horizontal axis in mm, and in mm^2^ for its absolute surface.

### In vivo MRI study

#### Patients selection

MRI data were retrospectively collected from electronic archiving database (PACS); the analysis included 6 female and 6 male adult patients, aged 43–71 years, with a mean BMI of 28.96 ± 12.09 (range 18.10–46.90) for women and 27.73 ± 3.90 (range 21.30–31.50) for men. Subjects were randomly selected from a large clinical database of patients having undergone a standardized head and neck MRI examination protocol. This was done under the agreement of the Bioethics committee of Cliniques Universitaires Saint-Luc (reference 2016/11mai/208).

#### MR equipment and parameters

**Fig. 2:**
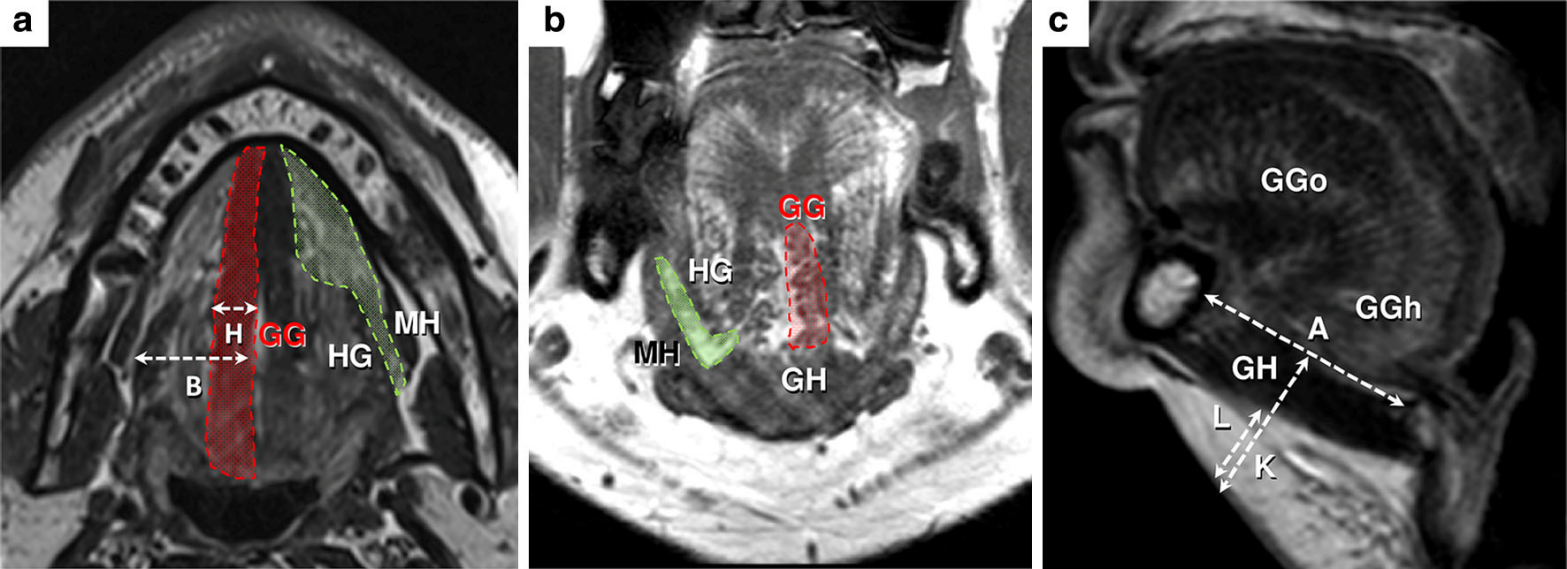
MRI study landmarks of the oral floor. **a** Axial section (T2-weighted). **b** Coronal section (T1-weighted). **c** Sagittal section (T2-weighted). Anatomical structures are labeled as follows: mylohyoid (*MH*), hyoglossus (*HG*), geniohyoid (*GH*), genioglossus (*GG*), horizontal fibers of GG (*GGh*), oblique fibers of GG (*GGo*). A single genioglossus area is highlighted in *red*. Sublingual regions are highlighted in *green*. Measurements were realized as indicated by *white arrows*: mandible-hyoid (*A*), skin to GG muscles (*K*) and skin to GH inferior surface (*L*) distances (color figure online)

All examinations were performed on the same 3 T MRI system equipped with 80 mT/m gradients (Achieva^®^ 3T, Philips HealthCare^®^, Best, The Netherlands) using a 16-channels Sense Head &Neck coil. Acquisition parameters were as follows: (1) for axial T2-weighted sequence (Fig. 2a): time of repetition (TR) 2936 ms; TE 65 ms; fast spin echo (FSE) mode with echo train length ETL 11; SF 1.7; NSA 1; slice thickness 4 mm with interslice gap 0.4 mm (10%); 26 slices for AT 2 min 56 s; (2) for coronal T1-weighted sequence (Fig. 2b), TR 649 ms; TE 9 ms; FSE mode with (ETL) 3; parallel imaging with sense (sensitivity encoding) factor (SF) 1.4; flow compensation (FC) option: on; fat suppression (FS) option: off; number of signal averaged (NSA) 1; slice thickness 4 mm with interslice gap 0.4 mm (10%); 26 slices for acquisition time (AT) 2 min 44 s; (3) for sagittal T2-weighted sequence (Fig. 2c): TR 1878 ms; TE 80 ms; FSE mode with ETL 15; SF none; NSA 1; slice thickness 5 mm with interslice gap 0.5 mm (10%); 26 slices for AT 1 min 08 s.

#### Images processing

OsiriX^®^ software [15] was used for DICOM images analysis. The neck circumference (NC) was measured horizontally just under the laryngeal prominence. The findings regarding morphometric measurements of the GG muscles and the distance from skin surface were compared to those obtained from cadaveric dissections.

### Statistical analysis

Morphometric data were expressed as mean ± SD. All statistical analyses were performed with Graphpad Prism^®^ software. A paired Student’s *t* test was used to compare the left and right sides. In the absence of difference between both sides, the left and right values of length were averaged on a per-subject level. To assess differences between males and females, we realized an unpaired Student’s *t* test. Unpaired Student’s *t* tests were also used to compare values between embalmed and fresh cadaveric heads dissections, and between cadaveric dissections and MRI study values. A significant *p* value was set at *p* < 0.05.

## Results

### Nerve dissection

In its distal submandibular course, the HGN gave rise to serial systematic branches supplying successively HG and GH muscles, before splitting into the terminal branches supplying the GG. No significant difference was found between the left and right sides regarding the number, the topography and the distribution of these branches.

### Branching pattern

**Fig. 3:**
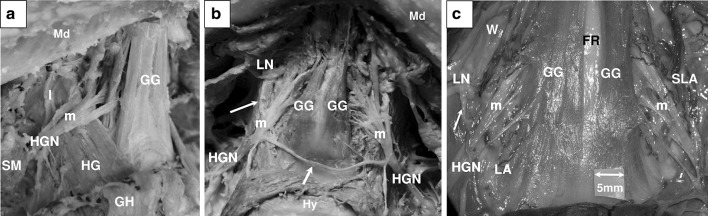
Nerve supply of genioglossus muscle. Photographs from dissections. **a** Oblique view of the right distal HGN and its adjacent structures in an embalmed specimen. **b** Antero-inferior view of the oral floor of an embalmed specimen. **c** Antero-inferior view of a *blue latex* injected fresh specimen under ×4.5 magnification loupe. Anatomical structures are labeled as follows: mandible (*Md*), hyoid bone (*Hy*), hypoglossal nerve (*HGN*), lateral contingent of the distal hypoglossal nerve (*l*), medial contingent of the distal hypoglossal nerve (*m*), genioglossus (*GG*), reclined geniohyoid (*GH*), hyoglossus (*HG*), lingual nerve (*LN*), submandibular gland (*SM*), submandibular Wharton’s duct (*W*), lingual artery (*LA*), sublingual artery (*SLA*), fatty raphe (*FR*). A communicating nervous fascicle between both hypoglossal nerves was observed in one embalmed subject and is indicated by the *yellow arrow*. An anastomotic fascicle between the HGN and the LN is pointed by the *white arrows*

Following the lateral surface of the HG, the HGN supplied 2.9 ± 0.6 collaterals (l-HGN) for that muscle. The distal portion of the nerve then gave off 1.2 ± 0.4 branches to the GH muscle. At the anterior edge of HG muscle, the m-HGN turned medially and spread out into terminal branches to enter forward the GG muscle (Fig. 3a). In 24 out of 30 dissected anatomic specimens, we could observe 1.3 ± 0.6 primary nerve branches that ran specifically into the GGh compartment. In these specimens, the m-HGN gave off 3.6 ± 0.7 first order branches supplying the GGo compartment, before penetrating the muscle bellies. In the 6 specimens without specific primary branch penetrating GGh, the m-HGN provided 3.5 ± 0.8 common primary branches to the GG muscle. Some connections between the lingual nerve (LN) and HGN could be observed in several subjects. One branch ascending along the HG anterior edge and forming anastomoses with the LN could usually be observed in 15 dissected specimens (83% of cases) (Fig. 3b, c). In one embalmed subject, we observed an anastomotic branch between both HGN, crossing the midline in the soft plan between GH and GG muscles (Fig. 3b).

### Morphometry

To assess the topography of the GG motor nerve supply, several measurements were realized. As no statistical difference between the left and right sides was observed, present data were expressed as the mean of both side data for each subject. No significant difference was noticed between men and women. All measurements are reported in Table 1. First of all, the hyoid-to-mandible distance (*A*) was found 44.1 ± 5.0 mm for embalmed cadavers and 55.6 ± 3.0 mm for fresh heads (*p* < 0.05).

**Table 1:** Morphometric measurements from dissections and in vivo MRI study

	Embalmed subjects (*n* = 17)	Fresh heads (*n* = 5)	MRI study (*n* = 12 patients)
*A* Mandible—hyoid	44.1 ± 5.0 (38–54)	55.6 ± 3.0 (52–60)^a^	44.05 ± 5.45 (36.23–55.03)^c^
*B* HGN when crossing over the HG anterior edge from the midline	17.7 ± 3.3 (12–24)	16.0 ± 1.0 (15–18)	15.65 ± 3.48 (10.42–22.76)^b^
*C* HGN spreading selective branches for GG from the mandible	35.7 ± 4.3 (27–43)	28.3 ± 5.0 (19–33) for GGo 39.5 ± 1.0 (38–40) for GGh	
*D* HGN supra-selective branches for GGh from the hyoid	21.3 ± 3.0 (14–25)	17.8 ± 4.0 (12–25)^a^	
*E* HGN supra-selective branches for GGo from the hyoid	27.3 ± 3.0 (20–31)	27.8 ± 6.0 (23–36)	
*F* Depth of HGN selective branches for GGh from the inferior surface of GG	3.8 ± 1 (2–6)	2.3 ± 1 (2–3)	
*G* Depth of HGN terminal branches for GGo from the inferior surface of GG	7.8 ± 1.1 (6–9)	8.7 ± 1 (7–10)	
*H* Width of single GG muscle	9.2 ± 1.3 (6.5–12)	10.2 ± 3.0 (7–14)	8.23 ± 0.86 (6.9–9.9)^b, c^
*I* Width of the fatty raphe	2.4 ± 1.1 (1–5)	2.7 ± 1.0 (1.5–5)	2.15 ± 0.79 (1.17–3.36)
*J* Width of both GG muscles	20.7 ± 2.9 (15–27)	21.4 ± 5 (16–28)	18.26 ± 2.0 (15.01–21.7)^b, c^
*K* Skin—GG muscles	27.3 ± 3.1 (20–30)	28.0 ± 4.0 (24–32)	28.42 ± 5.45 (20.47–38.94)
*L* Skin—GH muscles inferior surface			15.66 ± 5.38 (10.03–28.32)

Means, SD and ranges in mm

Capital letters refer to measured distances illustrated in Fig. 1

^a^ *p* < 0.05 for the unpaired Student’s *t* test comparing fresh and embalmed subjects data

^b^ *p* < 0.05 for the unpaired Student’s *t* test comparing MRI and embalmed subjects data

^c^ *p* < 0.05 for the unpaired Student’s *t* test comparing MRI and fresh subjects data

Distances *B* to *G* were used to localize the distal HGN and its selective branches for the GG muscle. The m-HGN was identified when it crossed the HG anterior edge (*B*) at 17.7 ± 3.3 and 16.0 ± 1.0 mm from the midline for embalmed and fresh specimens, respectively (*p* > 0.05). In embalmed specimens, the m-HGN selective branches for the GG muscle were found at 35.7 ± 4.3 mm from the mandible (*C*). In fresh heads, distinct distances were measured from the mandible to localize the m-HGN selective primary branches for the GGh (39.5 ± 1.0 mm) and for the GGo (28.3 ± 5.0 mm). Supra-selective m-HGN branches for GGh (*D*) were found at 21.3 ± 3.0 and 17.8 ± 4.0 mm from the hyoid lesser cornu for embalmed and fresh specimens, respectively (*p* < 0.05), while for the supra-selective branches for GGo (*E*), they were found at 27.1 ± 3.5 and 27.8 ± 6.0 mm, respectively (*p* > 0.05). The macroscopic muscular junctions of the m-HGN branches supplying the GGh were 3.8 ± 1.0 mm deep from the GG inferior surface (*F*) in embalmed and 2.3 ± 1.0 mm deep in fresh specimens (*p* > 0.05), while the depths of m-HGN branches junctions supplying the GGo (*G*) were 7.8 ± 1.1 and 8.7 ± 1.0 mm, respectively (*p* > 0.05). The terminal HGN diameter was measured under the digastric tendon and alongside the HG muscle at 2.6 ± 0.6 mm in embalmed and 2.3 ± 1.0 mm in fresh specimens (*p* > 0.05). Distances *H* to *K* were used to assess the GG muscle dimensions in the region of interest and its access. The transversal width of a single GG muscle in the nerve junction area (*H*) was measured at 9.2 ± 1.3 and at 10.2 ± 3.0 mm in embalmed and in fresh specimens, respectively (*p* > 0.05). The GG width of both muscles (*J*) was 20.7 ± 2.9 and 21.4 ± 5 mm (*p* > 0.05). They included the width of the fatty raphe between the GG muscles (*I*) (2.4 ± 1.1 and 2.7 ± 1.0 mm, respectively) (*p* > 0.05). Finally, the distance from the skin to the GG inferior surface was found to be 27.3 ± 3.1 mm in embalmed and 28.0 ± 4.0 mm in fresh specimens (*p* > 0.05) (*K*).

### In vivo MRI findings

No significant asymmetry between left and right sides was found (*p* > 0.05). No significant differences were noticed between men and women (*p* > 0.05). However, we noticed a trend for shorter geniohyoid distance (*A*) in women than in men with a mean difference of 5.35 ± 2.84 mm.

On coronal and transversal sections (Fig. 2a, b), neck circumference (NC) was found to be 45.3 ± 8.2 cm (range 34.3–58.0). The distance from the HG anterior edge to the midline (*B*) is 15.15 ± 4.81 mm (Table 1). The GG single muscle width (*H*) was 8.23 ± 0.86 mm which was significantly lower than the corresponding cadaveric data. The width of the fatty raphe (*I*) was 2.15 ± 0.79 mm. The distance between the lateral sides of each GG muscles in the region of nerve junctions (*J*) was 18.26 ± 2.00 mm. On sagittal sections (Fig. 2c), the distance between the mandible and the hyoid bone was found to be 44.05 ± 5.45 mm (*A*), which was significantly shorter than in fresh heads. The maximal length measured from the GG mandibular insertions to the dorsum of tongue was 52.38 ± 5.71 mm. Sagittal incidences allowed to precisely measure the depth of the submental adipose tissue. The skin to GG inferior surface distance (*K*) was found to be 28.42 ± 5.45 mm. The distance between the skin and the GH muscles inferior surface (*L*), equivalent to the subcutaneous adipose panicle, was measured at 15.66 ± 5.38 mm perpendicularly from the submental skin surface.

### Delineation of HGN selective stimulation area

**Fig. 4:**
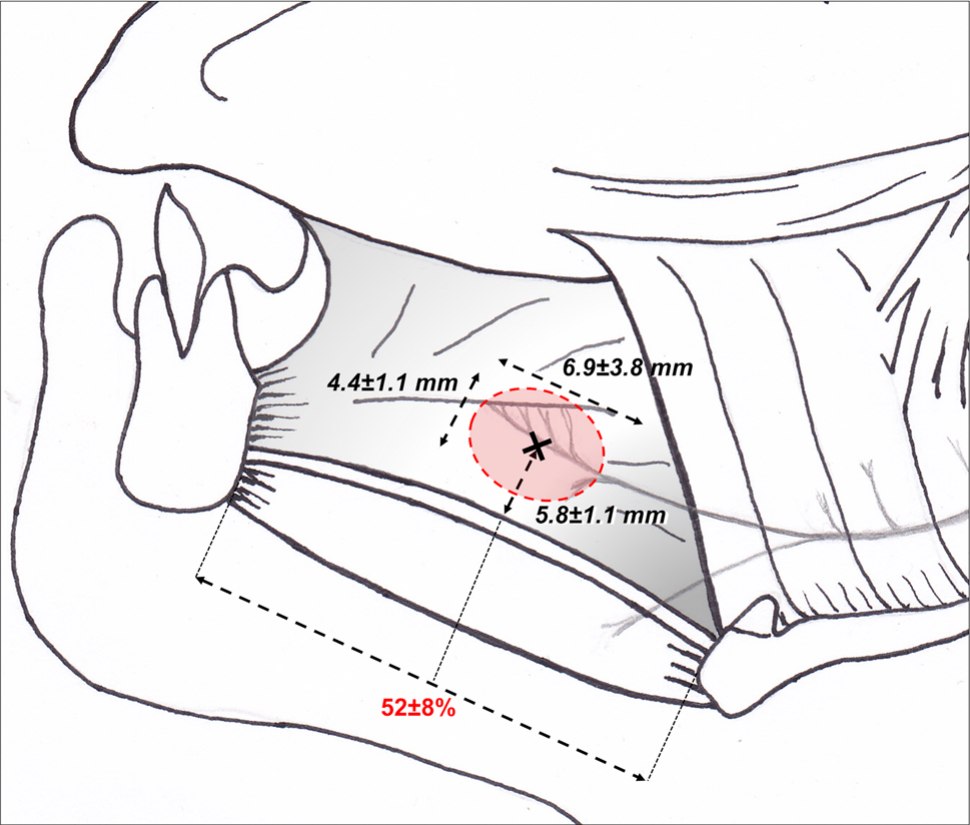
Delineation of HGN selective stimulation area. Starting from the hyoid bone, selective nervous branches for GGh and GGo were, respectively, localized at 44 ± 8 and 59 ± 8% of the geniohyoid distance, with a mean common central point (*black cross*) for both branches located roughly at the midpoint of anteroposterior axis (52 ± 8%) and 5.8 ± 1.1 mm from the inferior border of the GG muscle. The area of stimulation (*dotted line*) was represented by an ellipse of 4.4 ± 1.1 short axis and 6.9 ± 3.8 mm long axis, with an absolute surface (*red shadow area*) of 23.8 ± 3.3 mm^2^ (color figure online)

Starting from the hyoid bone, selective nervous branches for GGh and GGo were localized at 44 ± 8 and 59 ± 8%, respectively, of the geniohyoid distance with a mean common central point for both branches located at 52 ± 8% on the anteroposterior axis and 5.8 ± 1.1 mm from the inferior border of the GG muscle. The stimulation surface was represented by an ellipse of 4.4 ± 1.1 mm short axis (height) and 6.9 ± 3.8 mm long axis (length), with an absolute area of 23.8 ± 3.3 mm^2^ (Fig. 4).

## Discussion

### Correlation between anatomical results and electrophysiological studies

Our observations are in line with classical anatomical studies [10, 20]. The distinction between a lateral muscular group, comprising mostly tongue retrusors, and a medial muscular group, containing mainly tongue protrusors, each supplied by different HGN branches, has been described in previous works [10, 19, 20]. Moreover, it has been shown that the anterior and the posterior parts of the tongue are different in their anatomy and physiology. For example, the type and the metabolic properties of muscle fibers vary according to their location in the tongue. Posterior fibers, responsible for coarsely graded activities like those involved in swallowing and long-lasting activities required among others for keeping the airway patent, are more fatigue resistant than the anterior muscles fibers, responsible for fine, swift movements involved in speech and mastication [17, 24]. A specific compartmental organization of the human tongue muscles, especially of the genioglossus muscle, has also been demonstrated by Mu and Sanders [10]. According to them, the segregation between GGh and GGo compartments provides a morphological and functional basis to understand the tongue static and dynamic behavior. One of the main roles ascribed to the GG is the tongue protrusion which is prominently driven by the GGh and the posterior GGo fascicles [16]. Then, these muscular fibers should be stimulated to get an optimal therapeutic effect in OSA. Electrophysiological studies showed that directing stimulation to the protrusive part of the GG is likely to improve the flow-mechanical response, while the obliquely oriented anterior fibers can lead to tongue depression [3, 12]. Furthermore, these investigations clearly showed that the stimulation of the longitudinally oriented posterior fibers of the GG stabilizes the pharynx more efficiently than the anterior part.

In our study, a first primary branch of the m-HGN could be observed (in 35 out of 44 anatomic specimens) and was considered to supply the GGh. However, this small branch was not always observed and was very thin in some cases. To obtain an effective protractive effect by neurostimulation, that branch should be included by the stimulation but not alone. Thus, an effective stimulation should activate the longitudinal fibers of the GG and should not target only the anterior part of that muscle which depresses the tongue if activated selectively. However, we can also hypothesize that HGN stimulation would provide a better protraction effect if it is distal to the nerves branches supplying the retrusor muscles group (l-HGN). Therefore, the distances measured in our study can help to evaluate the nerve supply for the tongue muscular compartments of interest.

### Correlation between embalmed specimens and fresh heads dissections

Between embalmed and fresh dissected specimens, no significant difference was noticed in the distances from the midline to HGN up to the anterior edge of the HG muscle, in the GG muscles width, in the depth of the nervous branches supplying the GG, in the distance from the skin to the GG muscles and in the HGN diameter. However, the distance separating mandible to the hyoid bone was significantly longer in fresh cadaveric heads (55.6 ± 3.0 mm; range 52–60) than in embalmed cadavers (44.1 ± 5.0 mm; range 38–54) with a difference of means of 11.5 ± 2.4 mm (*p* < 0.05). This difference could be explained by a tissue relaxation found on thawed fresh heads, as the embalming process could be perceived as responsible for a slight tissular retraction. Significant differences (*p* < 0.05) were also noticed in distances to identify HGN terminal branches supplying the GGo and the GGh, but could be caused by the same kind of tissular alterations.

Likewise, dissection data and MRI results showed interesting differences. First, mandible-hyoid (*A*) was significantly longer in fresh heads than in embalmed specimens and MRI subjects, confirming the tissue relaxation in fresh cadavers. Second, the widths of single (*H*) and both (*J*) GG muscles, respectively, were lower in MRI subjects than in both embalmed and fresh cadaveric heads, whereas the width of the fatty raphe did not vary. These differences attest the effect of the GG muscle tonus on its size and shape. Therefore, this postmortem changes have to be taken into account for neurostimulator design and implantation site planning, and then for surgical application to living patients.

### Benefits and limits of in vivo MRI findings

In its distal course, the HGN has a diameter that is too small to be identified by MRI. However, the HGN distal branches can be indirectly depicted from concomitant veins courses. On the contrary, an MRI allows correct in vivo study of muscular complexes. Our results from embalmed cadavers dissections and in vivo findings showed no significant difference (*p* > 0.05) in the mandible-to-hyoid distance (*A*), the width of the fatty raphe (*I*) and the skin to GG inferior surface distance (*K*). The HGN follows vessels in the sublingual space, laterally to the HG and the GH–GG complex muscles (Fig. 3c), but although these vascular landmarks can be used to trace indirectly the nerve, MRI does not allow to precisely depict the HGN distal course. Nevertheless, MRI can be considered an effective tool to explore the anatomy of the oral floor and its deep structures, particularly to evaluate the GG muscles dimensions and distances between anatomic landmarks in in vivo subjects. Compared to dissections, anatomic landmarks included the mandible, the GH and GG muscles and the MH and HG muscles. They were especially useful for localizing the sublingual space which contains the distal HGN and the associated neighboring structures. The fatty content does explain the high signal intensity at T1-weighted MRI imaging. The submandibular duct and vessels course between the hyoglossus and mylohyoid muscles, and their positions can be located easily on MRI. However, this technique is not usual to precisely depict the nerves in this region because of the limited spatial resolution [8]. Therefore, cadaveric observations combined with the sublingual vascular structures localization allow predicting the distal course of the HGN from the anterior edge of the HG muscle to its ramifications into the GG muscles. Finally, MRI could be a very useful tool to assess patient anatomy prior to a device implantation and to follow up morphological muscular changes after implantation, like hypertrophy or atrophy, as well as fibrotic changes within muscle bellies and around the implanted device.

### Deductive surgical considerations

Different course patterns of submental and sublingual arteries have been described to supply the sublingual space [7]. Sublingual and lingual veins follow the distal HGN. The sublingual space is highly vascularized and cautious dissection is therefore necessary during distal HGN branches exposure, aiming to reduce the risk of vascular injury and incidence of haemorrhagic complications of implantation. The latter indeed might impair the subsequent efficiency of the neurostimulation protocol. The thickness of the submental adipose panicle then remains an important preoperative factor to consider, before device implantation. It can seem logical that surgical implantation of devices in that area would be more or less complicated according to this depth. In our study, the subjects were chosen randomly and do not represent a specific apneic patient cohort. However, no significant correlation between BMI, neck circumference and the width of the subcutaneous adipose panicle was found (*p* > 0.05), though obesity factors like highest BMI tended to correspond to the highest neck circumference and depth of subcutaneous adipose panicle. The distance between the submental skin surface and the region of interest does not change HGN topographic anatomy, but should be taken in account during a surgical access due to its limited exposure, and thus difficulty to target specific supra-selective GGh branches. It could significantly vary between patients, especially if they are obese or/and with an important neck circumference and may complicate a surgery of the region.

Actual stimulation devices implanted in the submandibular region or in the upper cervical region [1, 11] are responsible for nonselective truncular HGN stimulation. Consequently, protractor and retrusor muscles of one side are stimulated indifferently. To obtain a more homogenous and physiological contraction, bilateral selective neuromuscular stimulation of the GG muscles might target the sole protractive GG effect, with a subsequent synergistic release of the pharyngeal airway. It could also allow to reduce the need for high power neurostimulation. The medial, contiguous, location of both GG muscles as well as the topography and small size of their nervous hilum, particularly in GGh, as demonstrated in our anatomical study, could be suitable to design neurostimulation electrodes covering selectively and bilaterally the targeted area. However, these hypotheses should have to be confirmed in vivo.

## Conclusion

This study reports extensive data on the intramuscular HGN distribution, matching relevant measurements from cadaveric specimens with concordant in vivo imaging morphometric data. Furthermore, it provides a specific GG stimulation map, identifying a strong anatomical target area that could represent the ideal implantation site for selective muscle stimulation resulting in tongue protraction. This area is located midway between the hyoid bone and the mandibular symphysis. At surgical approach, after splitting the GH muscles superficially, the selective stimulation target is a patch area of about 4 × 7 mm, located at a depth of about 6 mm from the lower surface of the GG.
